# Prostate specific antigen testing is associated with men’s psychological and physical health and their healthcare utilisation in a nationally representative sample: a cross-sectional study

**DOI:** 10.1186/1471-2296-15-121

**Published:** 2014-06-17

**Authors:** Evelyn M Flahavan, Frances J Drummond, Kathleen Bennett, Thomas I Barron, Linda Sharp

**Affiliations:** 1 Department of Pharmacology & Therapeutics, Trinity College, University of Dublin, Dublin, Ireland; 2 National Cancer Registry Ireland, Building 6800, Airport Business Park, Cork, Ireland

## Abstract

**Background:**

Prostate cancer incidence has risen considerably in recent years, primarily due to Prostate Specific Antigen (PSA) testing in primary care. The objective of this study was to investigate associations between PSA testing and the psychological and physical health, and healthcare utilisation of men in a population where PSA testing is widespread.

**Methods:**

A cross-sectional study was carried out in a population-representative sample of men ≥50 years enrolled in The Irish Longitudinal Study on Ageing (TILDA). TILDA participants underwent structured interviews, health assessments and completed standardised questionnaires. Men were classified as ever/never having received a PSA test. Multivariate logistic regression (Odds Ratios (OR) and 95% Confidence Intervals (CI) was used to determine associations between PSA testing, and men’s psychological and physical health and healthcare utilisation.

**Results:**

This analysis included 3,628 men, 68.2% of whom ever had a PSA test. In adjusted analysis, men with sub-threshold depression were significantly less likely to have had a PSA test, (OR = 0.79, 95% CI 0.64-0.97). Likelihood of having a PSA test was inversely associated with anxiety, but this was not significant (OR = 0.79, 95% CI 0.57-1.09). Frailty (OR = 0.61, 95% CI 0.31-1.05) and eligibility for free primary care (OR = 0.63, 95% CI 0.52-0.77) were also inversely associated with PSA testing. Positive associations were observed between PSA testing and more chronic illnesses (OR = 1.11, 95% CI 1.05-1.19), more primary care visits (OR = 1.03, 95% CI 1.01-1.05) and preventative health practices, including cholesterol testing and influenza vaccination (OR = 1.35, 95% CI 1.13-1.60).

**Conclusions:**

Men’s psychological and physical health and their healthcare utilisation are associated with PSA testing in primary care. The association between poorer psychological health, in particular sub-threshold depression, and reduced likelihood of PSA testing in primary care requires further investigation. These findings may have wider implications for other cancer screening.

## Background

Prostate cancer incidence has increased in the last two decades, due to increasing prostate specific antigen (PSA) testing and subsequent prostate biopsy [1]. Results from two large randomised controlled trials, have not definitively answered the question regarding the benefit of PSA testing on mortality [2,3]. Despite this, the rate of PSA testing in Ireland is high [4] and the number of PSA tests performed continues to rise [5]. The majority of PSA tests originate in general practice [4,5]. This opportunistic testing has led to an increase in prostate cancer incidence, younger age at diagnosis and a shift towards more localized disease [5,6]. Increased prostate cancer detection has important consequences for men’s quality-of-life [7,8]. There is no consensus between guidelines internationally on the use of PSA testing [9,10] and PSA testing of asymptomatic men is not a national policy in Ireland [11,12]. It is therefore important to understand factors associated with PSA testing of men in general practice.

Psychological health negatively impacts breast, cervical and colorectal cancer screening [13,14]. Its impact on cancer screening in men [15] and on PSA testing has received little attention, however, and results have been conflicting due to small sample sizes and different measures used [16-18]. In addition, a small number of studies have recently reported that markers of healthcare utilisation influenced whether men have PSA tests and other cancer screening [17,19,20].

Our objective was to investigate, at the population level, associations between PSA testing and men’s psychological and physical health and their health services utilisation.

## Methods

### Setting

Ireland has a mixed public-private healthcare system. Approximately one-third of the population are eligible for the state-funded General Medical Services (GMS) Scheme, as determined by means-test and age [21], which entitles them to free General Practitioner (GP) and hospital visits and prescriptions for a small fee per item. GPs are reimbursed for GMS patients by the Health Services Executive. Approximately half the population have private health insurance (PHI). However, most insurance plans do not cover GP visits, and patients pay between €50 and 60 per visit.

### Study population

This study population consisted of males aged ≥50 years participating in wave 1 (2009–2011) of The Irish Longitudinal Study on Aging (TILDA) [22]. TILDA is a study of the health, lifestyle and financial situation of a population-representative sample of people aged ≥50 years involving Computer Aided Personal Interview (CAPI) in participants’ homes, a Self-Completion Questionnaires (SCQ) and comprehensive health assessment (HA) in one of two health centres. Where travel to health centres was unfeasible (~10% of participants), nurses performed the HA in participant’s homes (Additional file 1). TILDA was approved by the Faculty of Health Sciences Research Ethics Committee of Trinity College Dublin. Potential participants, who were unable to give consent due to dementia or cognitive impairment, were excluded.

### Outcome variable

The main outcome variable was ever having had a PSA test. Men were included if they gave a definitive answer to the CAPI question asking had they ever had “a PSA blood test to screen for prostate cancer”. Men who responded “don’t know” or declined to answer were excluded (n = 116).

### Covariates

#### Healthcare utilisation

The self-reported healthcare utilisation variables recorded were i) number of GP visits in the previous year; eligibility for GMS [23] (yes/no), ii) cholesterol testing (ever/never), iii) influenza vaccination (ever/never), and iv) number of regular medicines (prescription/other) including chronic cardio-preventative medication, statins, and aspirin (yes/no) classified using WHO ATC Classification.

Three scales were used to measure psychological health; depression was assessed using the Centre for Epidemiologic Studies Depression (CES-D) scale (scored as 0–7 not depressed; 8–15 sub-threshold; ≥16 case-level depression [24]); anxiety was assessed using the Hospital Anxiety and Depression Scale (HADS-A): (scored as 0–7 not anxious; 8–10 borderline; ≥11 case-level anxiety [25]); global cognitive function was assessed using the Mini Mental State Examination (MMSE; scored as 26–30 normal cognitive function; 20–25 mild cognitive impairment; <20 moderate cognitive impairment [26]). Mild and moderate cognitive impairment groups were combined because of the small number of men in the latter group. Participants for whom data was unavailable were classified as “unspecified” for each of these categories [22].

Men’s overall physical health was measured by summing the number of self-reported chronic illnesses from the following list: heart attack, heart failure, angina; stroke; diabetes; hypertension; high cholesterol; lung disease; asthma; cataracts; cancer; Parkinson's disease; peptic ulcer; arthritis; osteoporosis or hip fracture. Men taking medications in the WHO-ATC category G04C were classified as having been treated for Benign Prostatic Hypertrophy (BPH). Previous cancer diagnosis (yes/no) was identified separately. A frailty score was derived within TILDA from five measurements; self-reported weight-loss of ≥4.5 kg in the year pre-interview; weakness based on grip-strength; self-reported exhaustion; gait speed; and low physical activity. Other variables associated with frailty were: self-reported arthritis, joint replacement and osteoporosis (yes/no), hip or wrist fracture (ever/never). Subjective health status variables investigated included overall self-rated health, and self-rated emotional or mental health (excellent/very good, good, fair/poor).

Socio-demographic characteristics including age, marital status, work status, smoking status, highest educational level achieved and PHI status, were recorded at the CAPI.

### Statistical analysis

Univariate analyses (chi-square test, Wilcoxson rank-sum) were used to identify associations between covariates and ever having had a PSA test. Logistic regression was used to build a multivariate model of predictors of PSA testing. Analysis was conducted in two stages. Firstly, a core model was developed from socio-demographic, healthcare utilisation and health status variables previously associated with PSA testing (age, marital status, education, employment, smoking status, number of GP visits) and covariates with a p-value <0.1 in univariate analyses. Collinearity was addressed by including one of two potentially correlated variables (e.g. number of chronic illnesses, but not number of medicines). Covariates retained in the core model were: number of chronic illnesses, influenza vaccine, prior cancer diagnosis, treated for BPH, and GMS eligibility. In stage two, psychological and physical health measures were added separately to the core model, to assess their independent association with PSA testing.

Sensitivity analysis was performed to examine effects on multivariate risk estimates of PSA testing of excluding men who had a previous prostate cancer diagnosis (n = 93). Individual comorbidities were assessed for association with PSA testing in the core model, as an additional analysis.

TILDA data V 1-7-3 and STATA V 12 were used for analyses. Significance at p < 0.05 was assumed.

## Results

### Study population characteristics

**Figure 1:**
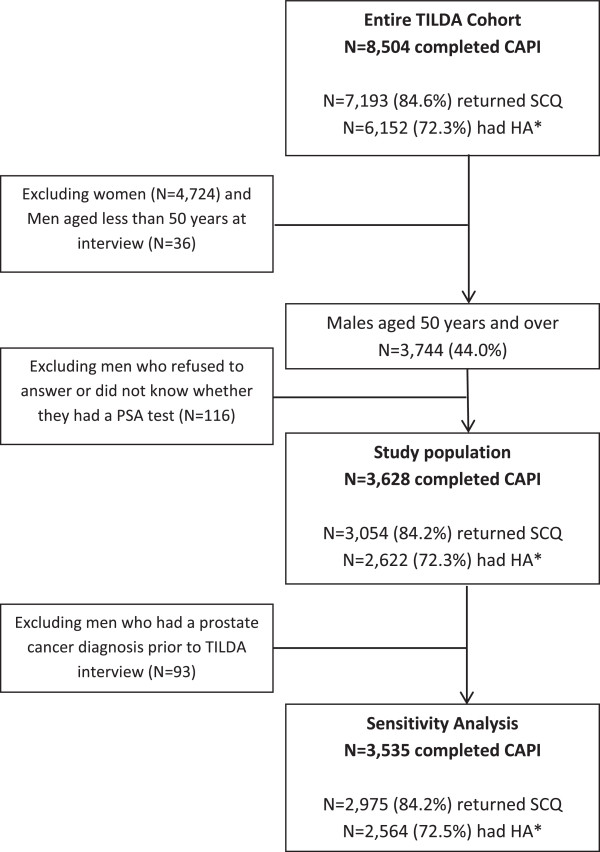
Flow diagram of the study population identified from the TILDA study.

The median age of men was 63 years (IQR 56–71, N = 3,628). 68.2% reported ever having a PSA test (Table 1). Of these men, 84.2% returned the SCQ, and HA data was available for 72.3% (Figure 1).

**Table 1:** Characteristics of the study population, stratified according to whether they ever or never had a PSA test (N (%), unless otherwise stated)

**Population characteristics**			**PSA tested**		
			**Ever**	**Never**	
			**N = 2,473 (68.2%)**	**N = 1,153 (31.8%)**	**p-value**
**Socio-demographic characteristics**					
Age at interview	Years	Median, (IQR)	64 (57, 71)	59 (54, 69)	<0.001
Marital status	Married	N (%)	1,916 (77.4)	795 (69.0)	<0.001
	Single	N (%)	248 (10.0)	178 (15.4)	
	Sep/Divorced	N (%)	112 (4.5)	89 (7.7)	
	Widowed	N (%)	199 (8.0)	91 (7.9)	
Education	Primary	N (%)	771 (31.2)	425 (36.9)	<0.001
	Secondary	N (%)	952 (38.5)	463 (40.2)	
	Third Level	N (%)	752 (30.4)	265 (23.0)	
Employment	Employed	N (%)	1,007 (40.7)	508 (44.1)	<0.001
	Retired	N (%)	1,225 (49.5)	406 (35.2)	
	Other	N (%)	243 (9.8)	239 (20.7)	
Smoking status	Never	N (%)	919 (37.2)	374 (32.4)	<0.001
	Past	N (%)	1,215 (49.1)	471 (40.8)	
	Current	N (%)	340 (13.7)	308 (26.7)	
PHI	At time of CAPI	N (%)	1,618 (65.4)	514 (44.6)	<0.001
**Health-care utilisation**					
No. of GP visits	year pre-CAPI	Median (IQR)	3 (1,5)	2 (0, 4)	<0.001
Cholesterol test	Ever	N (%)	2,408 (97.4)	791 (68.9)	<0.001
Influenza Vaccine	Ever	N (%)	1,352 (54.6)	468 (40.6)	<0.001
No. of medicines	Self-reported	Median (IQR)	2 (0, 4)	1 (0, 3)	<0.001
	BPH-Medicine	N (%)	166 (4.7)	21 (1.8)	<0.002
	Aspirin	N (%)	673 (27.2)	216 (18.7)	<0.001
	Statin	N (%)	869 (35.1)	277 (24.0)	<0.001
GMS eligibility	At time of CAPI	N (%)	1,072 (43.3)	544 (47.2)	0.029
**Physical, mental and emotional health**					
Overall health^1^	Excellent/Very good	N (%)	1,398 (56.5)	601 (52.3)	0.012
	Good	N (%)	690 (27.9)	376 (32.7)	
	Fair/Poor	N (%)	386 (15.6)	173 (15.0)	
No. chronic illnesses		Median (IQR)	2 (1, 3)	1 (0,2)	<0.001
Cancer diagnosis	Ever	N (%)	179 (7.2)	24 (2.1)	<0.001
	Prostate cancer	N (%)	93 (3.8)	0 (0)	<0.001
Frailty	Not frail	N (%)	1,265 (51.1)	486 (42.2)	<0.001
	Pre-frail	N (%)	481 (19.5)	244 (21.2)	
	Frail	N (%)	57 (2.3)	23 (2.0)	
	Unrecorded	N (%)	670 (27.1)	400 (34.7)	
Emotional/Mental health^1^	Excellent/Very good	N (%)	1,604 (64.8)	689 (59.8)	0.007
	Good	N (%)	676 (27.3)	347 (30.1)	
	Fair/Poor	N (%)	195 (7.9)	117 (10.2)	
Depression score	CESD	Median (IQR)	3 (0, 6)	3 (1, 8)	0.001
Depression	No	N (%)	1,954 (79.9)	847 (74.6)	0.002
	Sub-threshold	N (%)	344 (14.1)	200 (17.6)	
	Case-Level	N (%)	151 (6.2)	88 (7.8)	
Anxiety score	HADS-A (SCQ)	Median (IQR)	4 (2, 7)	5 (2, 7)	0.167
Anxiety categorical	Not anxious	N (%)	1,679 (67.8)	695 (60.2)	0.001
	Borderline	N (%)	277 (11.2)	115 (10.0)	
	Case-Level	N (%)	118 (4.8)	75 (6.5)	
	Unclassified	N (%)	401 (16.2)	268 (23.2)	
Cognition	MMSE score	Median (IQR)	29 (27, 30)	29 (28, 30)	
MMSE categorical	Normal	N (%)	1,677 (67.8)	699 (60.6)	<0.001
	Mild /moderate Impairment	N (%)	157 (6.3)	83 (7.2)	
	Unrecorded	N (%)	641 (25.9)	371 (32.2)	

^1^Self-rated overall and emotional or mental health relative to others of the same age; PHI: Private Health Insurance; CAPI: Computer Aided Personal Interview; SCQ: self-completed questionnaire, IQR: Interquartile range.

### Stage 1: Core model

The core multivariate model is presented in Table 2. Ever having a cholesterol test was the factor most strongly associated with PSA testing in univariate analysis (OR = 17.0 95%CI 12.9-22.4). Therefore, to assess other independent associations with PSA testing, this variable was removed from the model. In multivariate analyses, physical health (more chronic conditions (OR = 1.11 per unit increase in conditions, 95%CI 1.05-1.19); previous cancer diagnosis (OR = 2.74, 95%CI 1.74-4.30); BPH treatment (OR = 2.66, 95%CI 1.65-4.27)), healthcare utilisation (increased number of GP visits (OR = 1.01, 95%CI 1.01-1.05; having an influenza vaccination (OR = 1.35, 95%CI 1.13-1.60)); and socio-demographic variables (higher educational attainment and being married/cohabiting compared to other marital status) were associated with increased likelihood of having PSA tests. Men were significantly less likely to have had PSA tests if they were: current smokers (OR = 0.56, 95%CI 0.45-0.69), GMS eligible (OR = 0.63, 95%CI 0.52-0.77) or were not employed (OR = 0.67, 95%CI 0.53-0.85) (Table 2).

**Table 2:** Univariate (OR 95% CI) and multivariate (OR 95% CI) analysis of associations between socio-demographic characteristics, healthcare utilisation, and physical health, and ever having had a PSA test (Stage 1: Core Model)

**Variables associated with PSA testing**		**Univariate analysis**			**Multivariate analysis**		
		**OR**	**95% CI**	**p-value**	**OR**	**95% CI**	**p-value**
**Socio-demographic factors**							
Age at interview	Years	1.03	1.02-1.04	<0.001	1.02	1.00- 1.03	0.012
Marital status	Married	1.00	Ref		1.00	Ref	
	Single	0.58	0.47-0.72	<0.001	0.69	0.55-0.87	0.001
	Separated/Divorced	0.52	0.39-0.70	<0.001	0.68	0.50-0.92	0.014
	Widowed	0.91	0.70-1.18	0.466	0.70	0.53-0.94	0.017
Education	Primary	1.00	Ref		1.00	Ref	
	Secondary	1.13	0.96-1.33	0.131	1.31	1.09-1.57	0.004
	Third Level	1.56	1.30-1.88	<0.001	1.49	1.22-1.83	<0.001
Employment	Employed	1.00	Ref		1.00	Ref	
	Retired	1.52	1.30-1.78	<0.001	1.23	0.99-1.53	0.056
	Other	0.51	0.42-0.63	<0.001	0.67	0.53-0.85	0.001
Smoking status	Never	1.00	Ref		1.00	Ref	
	Past	1.05	0.89-1.23	0.553	0.96	0.81-1.14	0.634
	Current	0.45	0.37-0.55	<0.001	0.56	0.45-0.69	<0.001
Private health insurance	Yes	2.35	2.04-2.71	<0.001			
**Healthcare utilisation factors**							
Number of GP visits	Continuous	1.04	1.02-1.06	<0.001	1.03	1.01-1.05	0.001
Influenza vaccine	Ever	1.76	1.53-2.03	<0.001	1.35	1.13-1.60	0.001
GMS scheme eligible	Yes	0.86	0.74-0.98	0.029	0.63	0.52-0.77	<0.001
Number of medicines	Continuous	1.12	1.08-1.15	<0.001			
Cholesterol test	Yes	17.00	12.9-22.4	<0.001			
**Physical health**							
Chronic illnesses	Continuous	1.25	1.19-1.32	<0.001	1.11	1.05-1.19	0.001
Prior cancer diagnosis	Yes	3.66	2.38-5.64	<0.001	2.74	1.74-4.30	<0.001
Treated BPH	Yes	3.87	2.45-6.14	<0.001	2.66	1.65-4.27	<0.001

Variables for which multivariate ORs are presented are those contained within the core model; Multivariate ORs are adjusted for age (continuous), marital status (married/single/separated or divorced/widowed), highest education level attained (primary/secondary/third level), employment status (employed/retired/other), smoking status (never/past/current), number of GP visits in the past year (continuous), receipt of influenza vaccine (ever/never), number of chronic illness reported (continuous), GMS eligibility (yes/no), prior cancer diagnosis (yes/no) and reported receipt of medicines for BPH.

### Stage 2: associations between psychological and physical health and PSA testing

One-fifth of these men had depression, of whom 15% and 7% had sub-threshold and case-level depression, respectively. Prevalence of borderline and case-level anxiety were 16.3% and 5.4%, respectively. Men with sub-threshold depression were significantly less likely to have had a PSA test(s) (adjusted OR = 0. 79, 95%CI 0.62-0.97). Men with case-level anxiety had reduced likelihood of PSA testing in unadjusted analyses compared to non-anxious men, but this was not significant following adjustment (OR = 0.79, 95%CI 0.57-1.09). Lower self-rated emotional or mental health was associated with reduced likelihood of PSA testing in univariate analysis, but this was no longer significant in adjusted analyses (Table 3).

**Table 3:** Univariate (OR 95% CI) and multivariate (OR 95% CI) analysis of associations between psychological and physical health covariates, and ever having had a PSA test (Stage 2)

**Physical, mental and emotional health**		**Univariate analysis**			**Multivariate analysis**		
		**OR**	**95% CI**	**p-value**	**OR**	**95% CI**	**p-value**
**Self-rated health**	Excellent/Very good	1.00	Ref		1.00	Ref	
	Good	0.79	0.67-0.92	0.003	0.78	0.66-0.93	0.005
	Fair/Poor	0.96	0.78-1.18	0.688	0.88	0.69-1.38	0.320
**Self-rated emotional/mental health**	Excellent/Very good	1.00	Ref		1.00	Ref	
	Good	0.84	0.72-0.98	0.026	0.91	0.77-1.08	0.258
	Fair/Poor	0.72	0.56-0.92	0.008	0.82	0.62-1.08	0.158
**Depression**	CES-D	0.98	0.97-0.99	0.002	0.99	0.98-1.00	0.126
**Depression**	No	1.00	Ref		1.00	Ref	
	Sub-threshold	0.75	0.62-0.90	0.003	0.79	0.64-0.97	0.025
	Case-level	0.74	0.56-0.98	0.035	0.85	0.62-1.15	0.293
**Anxiety**	Continuous	0.98	0.96-1.00	0.078	1.00	0.97-1.02	0.791
**Anxiety categorical**	Not anxious	1.00	Ref		Ref	1.00	
	Borderline	0.99	0.79-1.26	0.980	1.02	0.79-1.30	0.906
	Case-level	0.65	0.48-0.88	0.003	0.79	0.57-1.09	0.159
	Unclassified	0.62	0.52-0.74	<0.001	0.71	0.59-0.87	0.001
**Cognition: MMSE score**	Continuous	1.04	1.01-1.08	0.023	1.05	1.01-1.10	0.024
**MMSE score for cognitive impairment**	Unimpaired	1.00	Ref		1.00	Ref	
	Mild-moderate	0.79	0.60-1.04	0.096	0.79	0.58-1.08	0.134
	Unrecorded	0.72	0.62-0.84	<0.001	0.84	0.71-1.00	0.049
**Frailty**	Not frail	1.00	Ref		1.00	Ref	
	Pre-frail	0.76	0.62-0.91	0.003	0.68	0.56-0.83	<0.001
	Frail	0.95	0.58-1.56	0.846	0.61	0.35-1.05	0.072
	Unrecorded	0.65	0.55-0.76	<0.001	0.72	0.60-0.85	<0.001
**Arthritis**		1.59	1.32-1.91	<0.001	1.23	0.99-1.52	0.058
**Aspirin**		1.62	1.36-1.92	<0.001	1.18	0.96-1.44	0.113
**Statin**		1.71	1.46-2.00	<0.001	1.28	1.06-1.54	0.009

Multivariate ORs are adjusted for the core model: adjusted for age (continuous), marital status (married/ single/separated or divorced/widowed), education level attained (primary/secondary/ third level), employment status (employed/retired/other), smoking status (never/ past/current), number of GP visits in the past year (continuous), receipt of influenza vaccine (ever/never), number of chronic illness reported (continuous), GMS eligibility (yes/no) prior cancer diagnosis (yes/no) and reported receipt of medicines for BPH.

Patients with a degree of cognitive impairment were significantly less likely to have had PSA tests. Those with mild-moderate cognitive impairment were less likely to have had PSA tests, compared to those with unimpaired cognition, though non-significantly (OR = 0.79, 95%CI 0.58-1.08).

Frailty was associated with reduced likelihood of PSA testing, this was significant for men who were pre-frail (adjusted OR = 0.68, 95%CI 0.56-0.83). Individual frailty measures associated with non-testing were low grip strength (OR = 0.84, 95%CI 0.69-1.02), low gait speed (OR = 0.61, 95%CI 0.43-0.86) and low levels of physical activity (OR 0.66, 95%CI 0.50-0.87) (Additional file 2). Men who reported heart attack/heart failure/angina (OR = 0.62, 95%CI 0.47-0.80), stroke (OR = 0.55, 95%CI 0.32-0.95) and lung disease (OR = 0.64, 95%CI 0.43-0.95) were significantly less likely to have had PSA tests in adjusted analyses (Additional file 3).

Exclusion of men who had a prostate cancer diagnosis (N = 93) did not affect associations between any covariates in the core model and ever having a PSA test, except previous cancer diagnosis (Additional file 4).

## Discussion

PSA testing is widespread in Ireland. However, our findings suggest that men with lower self-reported physical and psychological health, including depression, anxiety, cognitive impairment and frailty were less likely to have had a PSA test, while men with very good self-reported health were more likely to have had a PSA test, in this nationally representative sample of men aged 50 years or older, after adjusting for socio-demographic factors. Increased healthcare utilisation was also associated with increased likelihood of PSA testing, however men who were eligible for free healthcare were less likely to have been tested.

These results should be interpreted with some care, as the study was cross-sectional in design; however we considered three hypotheses of health behaviour to explain these observations [27,28]. Firstly, there is evidence of a ‘healthy user effect’ [27] whereby men taking preventative medication e.g. statins and receiving influenza vaccinations were more likely to have had PSA tests. The healthy user effect is a multidimensional concept incorporating ‘health-seeking’ tendencies, i.e. healthier patients request or accept more screening tests and have increased adherence to medications, but it also incorporates ‘health status’ i.e. the ability of patients, physically and cognitively to attend primary care and to get prescriptions filled [27]. Multi-morbidity results in polypharmacy and increased health services utilisation [29]. We found that, despite adjusting for number of GP visits, men with more chronic illnesses were more likely to have been tested, suggesting that some PSA tests can be ascribed to the ‘surveillance hypothesis’ i.e. men with coexisting conditions have more frequent contact with the healthcare system facilitating early diagnosis [20,28]. However, while not the central focus of this paper, we found that the association between comorbidity and PSA testing depended on the coexisting disease, which was in agreement with other studies [30]. In this cohort, likelihood of PSA testing was increased in men with angina, high cholesterol, cataracts and hypertension (‘surveillance hypothesis’), but was negatively associated with frailty, cardiac diseases and stroke, suggesting that poorer physical health may distract a GP from undertaking, or offering men a PSA test, the ‘competing demand hypothesis’ [28].

The negative associations observed, in this cohort, between poorer psychological health and likelihood of PSA testing are further evidence of the ‘competing demand hypothesis.’ The impact of poor psychological health on the likelihood of men having a PSA test has received little attention. Men with sub-threshold depression were significantly less likely to have PSA tests than men who were not depressed, suggesting that somatic symptoms associated with depression may be more pertinent during healthcare visits, or that GPs may be less likely to initiate discussions about PSA testing and prostate cancer with depressed men for fear of exacerbating their condition [31]. Our findings concur with previous work which observed lower rates of breast, cervical and colorectal cancer screening among people with depression [13,14,32], despite increased usage of primary care services [14]. However, we found that case-level depression was not associated with PSA testing. This may be due to various reasons; men with case-level depression may be receiving management for depression and thus may be more likely to be PSA tested, consistent with the ‘surveillance hypothesis’; the number of men with case-level depression may be too small to detect significant effects; or the effect of case-level depression on PSA testing may be no longer significant when other aspects of psychological health e.g. anxiety was included in the model. In support of the latter hypothesis, Kotwal *et al.* observed that men with depressive symptoms were less likely to have PSA tests however, this effect was mediated by levels of perceived stress [19] which concurs with the surveillance hypothesis. While stress was not measured in this cohort, we found that case-level anxiety was associated with reduced likelihood of PSA testing in univariate, but not adjusted analysis.

Anxiety has been shown to influence PSA testing in a number of ways. A review found variations in anxiety levels across the prostate cancer continuum from screening to beyond treatment [16]. Dale *et al.,* observed that PSA screening was associated with increased levels of anxiety in men, highest in those with high pre-dispositional anxiety, but this mostly subsided upon receipt of a normal result [16]. Furthermore, anxiety about having prostate cancer predicted both PSA testing and avoidance of screening, the latter especially among asymptomatic men with a family history of prostate cancer. To further elucidate the effect of anxiety on PSA testing Consedine *et al.*, in a small study of 533 American men, aged 45 to 70 years, investigated the effect of three components of anxiety i.e. dispositional anxiety, prostate cancer worry, and screening fear on frequency of PSA testing (and digital rectal exams (DRE)) [18]. They concluded that cancer worry propelled men to have PSA tests, screening fear deterred men from having DRE, but not PSA tests, and trait anxiety was associated with more frequent DREs, but not with PSA testing [18]. Furthermore, a recent study observed that the association between anxiety and PSA testing is dependent on the number of GP visits [19]; men were less likely to be PSA tested if they had higher anxiety and attended their GP once, but men with higher anxiety who attended their GP more frequently were more likely to be tested. However, it is difficult to draw comparisons between studies due to different study designs, populations examined and instruments used to measure psychological health (depression, anxiety and stress) [13-16,18,19].

Men with increased cognitive impairment were also significantly less likely to have had PSA tests, which again may be explained by the ‘competing demands’ hypothesis. This is the first time associations between psychological health and PSA testing has been observed in men in Ireland and our findings add to the growing body of literature on the effect of psychological health on preventative health and cancer screening.

Eligibility for free healthcare (GMS eligibility) is associated with more frequent GP visits [23], however, despite adjustment for socio-demographic, health, and healthcare factors including number of GP visits, GMS eligibility was negatively associated with PSA testing, which is consistent with income-related inequality in uptake of PSA testing observed in Ireland and elsewhere [33]. This highlights the issue that in mixed public-private systems, free healthcare services does not produce equity in uptake of primary care services, and may in part explain the higher prostate cancer incidence in higher socioeconomic groups [34].

Socio-demographic factors were strong predictors of PSA testing and our findings are broadly in agreement with others [19,20,33]. Married men were more likely to have PSA tests possibly because their wives engage in breast and cervical cancer screening (Drummond *et al.* unpublished data). Odds of PSA testing were greatly reduced in current smokers, this trend concurs with findings from previous studies [35]. Smoking-related illnesses may be prioritised by GPs, the ‘competing demands hypothesis’ and/or smokers may avoid engagement with health services because they anticipate unwanted advice to quit smoking [36].

This study has several strengths. It is a large sample, representative of the population [22], with data on a wide range of variables. Standardised measures of depression, anxiety and cognitive function were used, although stress was not measured. We acknowledge several limitations; data on PSA testing was self-reported, which is subject to recall bias [37]. However, that more than two-thirds of men reported ever having a PSA test is not surprising given the level of PSA testing in Ireland has risen since the mid 1990’s [38]; by 2004, 41.2% of men aged 50 years and older had had a PSA test [6], and the number of PSA tests continues to increase [5]. Sensitive information may have been withheld e.g. use of anti-depressants; or chronic conditions misclassified and the strength of some associations with PSA testing may have been limited due to small numbers in sub-groups. A small number of men in this study (n = 93; 2.6%) had a previous prostate cancer diagnosis, and the prevalence of psychological distress (including depression and anxiety) has been shown to be elevated in men with prostate cancer in some studies [39,40], but not others [41]. Analyses were adjusted for previous cancer diagnosis and a sensitivity analysis was conducted excluding men with a previous prostate cancer diagnosis (Additional file 4: Table S4). Finally, there is potentially residual or unmeasured confounding in the analysis for example the influence of GPs, or ‘provider effect’, on whether men were PSA tested.

## Conclusions

In conclusion, this cross-sectional study provides insight into the characteristics of men who have, and have not had PSA tests in primary care. Men in poorer psychological and physical health, smokers and those eligible for free GP services were less likely to have had PSA tests while men in good overall health and those engaging in health-seeking behaviours were more likely to have been tested. These findings might be considered by physicians and policy makers in the development of public health strategies to improve the appropriateness and equality of prostate cancer detection.

## Abbreviations

PSA: Prostate specific antigen; TILDA: The Irish longitudinal study on ageing; CAPI: Computer assisted personal interview; SCQ: Self completed questionnaire; HA: Health assessment; GMS: General medical services; OR: Odds ratio; CI: Confidence interval; GP: General practice; WHO ATC: World Health Organisation anatomical therapeutic chemical classification; BPH: Benign prostatic hypertrophy; CES-D: Centre for epidemiologic studies depression; HADS-A: Hospital anxiety and depression scale; PHI: Private health insurance; DRE: Digital rectal examination.

## Competing interests

Since the completion of this study EMF has been employed by Eli Lilly and Company. LS has previously received an unrestricted project grant from Sanofi-Aventis.

## Authors’ contributions

EMF participated in the study design, carried out the statistical analysis and drafted the manuscript. FJD participated in the study design and drafted the manuscript. KB, TIB and LS conceived of the study, participated in its design and commented critically on the draft manuscript. All authors contributed to interpretation of the results, and read and approved the final manuscript.

## Authors’ information

EMF: Irish Cancer Society Research Scholar in the Department of Pharmacology and Therapeutics TCD (2010–2013) – focusing on Prostate Cancer Pharmacoepidemiology; FJD, PhD is a research fellow/project coordinator at the National Cancer Registry; KB, PhD is an associate professor in Pharmacoepidemiology and statistician in the Department of Pharmacology and Therapeutics TCD; ITB, PhD, is a research fellow in the Department of Pharmacology and Therapeutics TCD; LS PhD, is an epidemiologist at the National Cancer Registry and adjunct professor, Department of Epidemiology and Public Health, UCC.

## Pre-publication history

The pre-publication history for this paper can be accessed here:

http://www.biomedcentral.com/1471-2296/15/121/prepub

## Supplementary Material

Additional file 1: Table S1 List of covariates captured by the TILDA study; variables (N (%)) from the TILDA study included in this study population, for univariate and multivariate analyses. Click here for file

Additional file 2: Table S2 Univariate (OR 95% CI) and multivariate (OR 95% CI) analysis of associations between covariates which make up the frailty score, and ever having had a PSA test. Click here for file

Additional file 3: Table S3 Post-hoc analysis; Univariate (OR 95% CI) and multivariate (OR 95% CI) analysis of associations between chronic illnesses and ever having had a PSA test. Click here for file

Additional file 4: Table S4 Assessment of the association between PSA testing (yes/no) and covariates associated with PSA testing having excluded men with prior prostate cancer. Click here for file
