# Supplementary material for: Prostate specific antigen testing is associated with men’s psychological and physical health and their healthcare utilisation in a nationally representative sample: a cross-sectional study
Source: BMC Fam Pract. 2014 Jun 17;15:121. doi: 10.1186/1471-2296-15-121 (PMC4065544; doi:10.1186/1471-2296-15-121)
Supplement: Additional file 1: Table S1 — List of covariates captured by the TILDA study; variables (N (%)) from the TILDA study included in this study population, for univariate and multivariate analyses. [file 1471-2296-15-121-S1.docx]

**Additional file 1: Detailed description of dataset covariates:**

**Table S1:** List of covariates captured by the TILDA study; variables (N (%)) from the TILDA study included in this study population, for univariate and multivariate analyses.

| **Dataset Covariates** | **Details** | **Variable type** | **Capture** | **Univariate** | **Multivariate** |
| --- | --- | --- | --- | --- | --- |
| **Socio-demographic Characteristics** |  |  |  | **N (%)** | **N (%)** |
| Age at interview | (years) | Continuous | CAPI | 3,624 (99.9%) | 3,624 (99.9%) |
| Marital Status |  | Categorical | CAPI | 3,628 (100%) | 3,624 (99.9%) |
| Education |  | Categorical | CAPI | 3,628 (100%) | 3,624 (99.9%) |
| Employment |  | Categorical | CAPI | 3,628 (100%) | 3,624 (99.9%) |
| Smoking Status |  | Categorical | CAPI | 3,627 (100%) | 3,624 (99.9%) |
| **Health-Care Utilisation** |  |  |  |  |  |
| Number of GP visits | year pre-CAPI | Continuous | CAPI | 3,628 (100%) | 3,624 (99.9%) |
| Cholesterol test | Ever/Never | Categorical | CAPI | 3,620 (99.8%) | n/a |
| Influenza Vaccine | Ever/Never | Categorical | CAPI | 3,628 (100%) | 3,624 (99.9%) |
| Chronic illnesses^1^ | Sum (from list) | Continuous | CAPI | 3,628 (100%) | 3,624 (99.9%) |
| Cancer diagnosis | Ever/Never | Categorical | CAPI | 3,628 (100%) | 3,624 (99.9%) |
| Number of medicines – WHO ATC code | Self-report | Continuous | CAPI | 3,595 (99.1%) | n/a |
| Aspirin – ( WHO ATC: B01AC06; M01BA03, N02BA01, N02BA51, N02BA71) | Self-report at CAPI | Categorical | Generated | 3,628 (100%) | 3,628 (100%) |
| Statin – (WHO ATC: C10AA) | Self-report at CAPI | Categorical | Generated | 3,628 (100%) | 3,628 (100%) |
| GMS Scheme Eligibility | At CAPI | Categorical | CAPI | 3,628 (100%) | 3,624 (99.9%) |
| Private Health Insurance | At CAPI | Categorical | CAPI | 3,628 (100%) | n/a |
| **Mental and Emotional Health** | | | |  |  |
| Self-rated Emotional or Mental Health | Likert | Categorical | CAPI | 3,628 (100%) | 3,624 (99.9%) |
| Depression Score | 8 item CESD | Continuous | HA | 2,610 (71.9%) | 2,608 (71.9%) |
| Depression | CESD | Categorical | CAPI | 3,584 (98.8%) | 3,581 (98.7%) |
| Anxiety Score | HADS-A | Continuous | SCQ | 2,959 (81.4%) | 2,956 (81.5%) |
| Anxiety Categorical | from HADS-A | Categorical | Generated^2^ | 3,628 (100%) | 3,624 (99.9%) |
| MMSE Score |  |  | HA | 2,616 (72.1%) | 2,614 (72.1%) |
| MMSE Categorical | from MMSE | Categorical | Generated^2^ | 3,628 (100%) | 3,624 (99.9%) |
| **Physical Health** |  |  |  |  |  |
| Self-rated health relative to others of the same age | Likert | Categorical | CAPI | 3,624 (99.9%) | 3,620 (99.8%) |
| Frailty score categories**^3^** |  | Categorical | HA | 2,556 (70.5%) | n/a |
| Frailty |  | Categorical | Generated^2^ | 3,628 (100%) | 3,624 (99.9%) |
| Weight Loss unintended of 4.5 kg or more | in past year | Categorical | CAPI | 3,619 (99.8%) | 3,615 (99.6%) |
| Low Grip Strength |  | Categorical | HA | 2,614 (72.1%) | 2,612 (72.0%) |
| Self-report exhaustion |  | Categorical | CAPI | 3,626 (99.9%) | 3,621 (99.8%) |
| Gait Speed |  | Categorical | HA | 2,590 (71.4%) | 2,207 (60.8%) |
| Low Activity (IPAQ <383 kcal for men) | 8 item IPAQ | Categorical | CAPI | 2,589 (71.4%) | 2,587 (71.3%) |
| Fracture hip or wrist |  | Categorical | CAPI | 3,628 (100%) | 3,529 (97.3%) |
| Fall in past year |  | Categorical | CAPI | 3,628 (100%) | 3,622 (99.8%) |
| Joint replacement |  | Categorical | CAPI | 3,627 (100%) | 3,623 (99.8%) |

^1^Chronic illnesses: sum from self-reported: heart attack or heart failure or angina; stroke; diabetes; self-reported hypertension; self-reported high cholesterol; lung disease; asthma; cataracts; cancer; Parkinson's disease; peptic ulcer; arthritis; osteoporosis or hip fracture.

^2^Generated categorical variables for anxiety, MMSE and Frailty were recorded where there were observations missing to give a complete dataset
^3^Frailty score categories: derived from five measurements (i) self-reported weight-loss, of 4.5kg (10 lb) or more in the year pre-interview (CAPI); (ii) weakness based on grip-strength (home assessment or health center); (iii) self-reported exhaustion (CAPI); (iv) gait speed (home assessment or health center); (v) low physical activity (International Physical Activity Questionnaire shortened form, within CAPI).
