# Supplementary material for: Prostate specific antigen testing is associated with men’s psychological and physical health and their healthcare utilisation in a nationally representative sample: a cross-sectional study
Source: BMC Fam Pract. 2014 Jun 17;15:121. doi: 10.1186/1471-2296-15-121 (PMC4065544; doi:10.1186/1471-2296-15-121)
Supplement: Additional file 2: Table S2 — Univariate (OR 95% CI) and multivariate (OR 95% CI) analysis of associations between covariates which make up the frailty score, and ever having had a PSA test. [file 1471-2296-15-121-S2.docx]

**Additional file 2:**

**Table S2:** Univariate (OR 95% CI) and multivariate (OR 95% CI) analysis of associations between covariates which make up the frailty score, and ever having had a PSA test

| **Frailty Covariates** | **PSA test** | |  | **Unadjusted Analysis** | | **Adjusted Multivariate** | |
| --- | --- | --- | --- | --- | --- | --- | --- |
|  | **Ever** | **Never** |  |  |  |  |  |
|  | **N (%)** | **N (%)** | **p-value** | **OR (95% CI)** | **p-value** | **OR (95% CI)** | **p-value** |
| **Weight Loss** | 155 (67.1) | 76 (32.9) | 0.691 | 0.94 (0.71-1.25) | 0.691 | 0.93 (0.67-1.27) | 0.629 |
| **Low Grip Strength** | 754 (70.8) | 311 (29.2) | 0.579 | 1.05 (0.88-1.25) | 0.579 | 0.84 (0.69-1.02) | 0.085 |
| **Self-report exhaustion** | 186 (63.3) | 108 (36.7) | 0.057 | 0.79 (0.61-1.01) | 0.057 | 0.83 (0.63-1.10) | 0.192 |
| **Gait Speed** | 154 (68.4) | 71 (31.6) | 0.504 | 0.90 (0.67-1.21) | 0.505 | 0.61 (0.43-0.86) | 0.005 |
| **Low Activity** | 190 (64.6) | 104 (35.4) | 0.025 | 0.74 (0.58-0.96) | 0.025 | 0.66 (0.50-0.87) | 0.003 |
| **Fracture hip or wrist** | 291 (68.5) | 134 (31.5) | 0.883 | 1.02 (0.82-1.26) | 0.883 | 1.07 (0.85-1.35) | 0.581 |
| **Fall in past year** | 453 (68.5) | 208 (31.5) | 0.840 | 1.02 (0.85-1.22) | 0.840 | 0.94 (0.77-1.14) | 0.534 |
| **Joint replacement** | 203 (77.5) | 59 (22.5) | 0.001 | 1.66 (1.23-2.23) | <0.001 | 1.33 (0.97-1.83) | 0.080 |

Multivariate OR is adjusted for age (continuous), marital status (married/ single/separated or divorced/ widowed), education level attained (primary/ secondary/ third level), employment status (employed/retired/ other), smoking status (never/ past/ current), number of GP visits in the past year (continuous), receipt of influenza vaccine (ever/never), number of chronic illness reported (continuous) , GMS eligibility (yes/no), prior cancer diagnosis (yes/no) and reported receipt of medicines for BPH.
