# Supplementary material for: Prostate specific antigen testing is associated with men’s psychological and physical health and their healthcare utilisation in a nationally representative sample: a cross-sectional study
Source: BMC Fam Pract. 2014 Jun 17;15:121. doi: 10.1186/1471-2296-15-121 (PMC4065544; doi:10.1186/1471-2296-15-121)
Supplement: Additional file 3: Table S3 — Post-hoc analysis; Univariate (OR 95% CI) and multivariate (OR 95% CI) analysis of associations between chronic illnesses and ever having had a PSA test. [file 1471-2296-15-121-S3.docx]

**Additional file 3:**

**Table S3:** Post-hoc analysis; Univariate (OR 95% CI) and multivariate (OR 95% CI) analysis of associations between chronic illnesses and ever having had a PSA test

| **Self-reported chronic Illness** | **PSA test** | |  | **Unadjusted Analysis** | | **Multivariate Analysis** | |
| --- | --- | --- | --- | --- | --- | --- | --- |
|  | **Ever** | **Never** |  |  |  |  |  |
|  | **N (%)** | **N (%)** | **p-value** | **OR (95% CI)** | **p-value** | **OR (95% CI)** | **p-value** |
| Heart attack/Heart Failure/ Angina | 298 (70.0) | 128 (30.0) | 0.413 | 1.1 (0.88-1.37) | 0.413 | 0.62 (0.47-0.80) | <0.001 |
| Angina | 188 (75.5) | 61 (24.5) | 0.011 | 1.47 (1.09-1.98) | 0.011 | 0.83 (0.59-1.16) | 0.266 |
| Heart attack | 179 (67.8) | 85 (32.2) | 0.880 | 0.98 (0.75-1.28) | 0.880 | 0.59 (0.44-0.80) | 0.001 |
| Heart failure | 42 (75.0) | 14 (25.0) | 0.272 | 1.40 (0.63-2.58) | 0.274 | 0.96 (0.50-1.83) | 0.892 |
| Stroke | 45 (66.2) | 23 (33.8) | 0.715 | 0.91 (0.55-1.51) | 0.715 | 0.55 (0.32-0.95) | 0.031 |
| Diabetes | 262 (73.6) | 94 (26.4) | 0.022 | 1.33 (1.04-1.71) | 0.022 | 0.96 (0.73-1.27) | 0.792 |
| Hypertension | 965 (72.6) | 364 (27.4) | <0.001 | 1.39 (1.19-1.61) | <0.001 | 0.94 (0.78-1.14) | 0.531 |
| High Cholesterol | 996 (75.5) | 324 (24.6) | <0.001 | 1.72 (1.48-2.00) | <0.001 | 1.51 (1.24-1.83) | <0.001 |
| Lung Disease | 89 (65.0) | 48 (35.0) | 0.404 | 0.86 (0.60-1.23) | 0.404 | 0.64 (0.43-0.95) | 0.027 |
| Asthma | 192 (69.8) | 83 (30.2) | 0.554 | 1.08 (0.83-1.42) | 0.554 | 0.83 (0.62-1.11) | 0.204 |
| Cataracts | 233 (74.0) | 82 (26.0) | 0.022 | 1.36 (1.04-1.76) | 0.023 | 0.83 (0.62-1.13) | 0.242 |
| Parkinson’s Disease | 19 (86.4) | 3 (13.6) | 0.067 | 2.97 (0.88-10.04) | 0.081 | 2.41 (0.67-8.73) | 0.179 |
| Peptic Ulcer | 212 (71.1) | 86 (28.9) | 0.258 | 1.16 (0.90-1.51) | 0.259 | 1.03 (0.78-1.38) | 0.813 |
| Arthritis | 588 (75.7) | 189 (24.3) | <0.001 | 1.59 (1.32-1.91) | <0.001 | 1.23 (0.99-1.52) | 0.058 |
| Osteoporosis | 56 (77.8) | 16 (22.2) | 0.079 | 1.65 (0.94-2.88) | 0.081 | 1.17 (0.65-2.10) | 0.598 |
| Hip Fracture | 106 (74.7) | 36 (25.4) | 0.094 | 1.39 (0.94-2.04) | 0.096 | 1.07 (0.71-1.60) | 0.760 |

Multivariate OR is adjusted for age (continuous), marital status (married/ single/separated or divorced/ widowed), education level attained (primary/secondary/third level), employment status (employed/retired/ other), smoking status (never/past/current), number of GP visits in the past year (continuous), receipt of influenza vaccine (ever/never), number of chronic illness reported (continuous) , GMS eligibility (yes/no), prior cancer diagnosis (yes/no) and reported receipt of medicines for BPH.
