# Supplementary material for: Prostate specific antigen testing is associated with men’s psychological and physical health and their healthcare utilisation in a nationally representative sample: a cross-sectional study
Source: BMC Fam Pract. 2014 Jun 17;15:121. doi: 10.1186/1471-2296-15-121 (PMC4065544; doi:10.1186/1471-2296-15-121)
Supplement: Additional file 4: Table S4 — Assessment of the association between PSA testing (yes/no) and covariates associated with PSA testing having excluded men with prior prostate cancer. [file 1471-2296-15-121-S4.docx]

**Additional file 4:**

Table S4: Assessment of the association between PSA testing (yes/no) and covariates associated with PSA testing having excluded men with prior prostate cancer.

| **Variables associated with PSA testing** | | **Univariate Analysis** | | | **Multivariate Adjusted** | | |
| --- | --- | --- | --- | --- | --- | --- | --- |
|  |  | **OR** | **95% CI** | **p-value** | **OR** | **95% CI** | **p-value** |
| Age at interview | years | 1.03 | 1.02-1.03 | <0.001 | 1.02 | 1.00- 1.03 | 0.013 |
| Marital Status | Married | 1.00 | Ref |  | 1.00 | Ref |  |
|  | Single | 0.58 | 0.47-0.71 | <0.001 | 0.69 | 0.55-0.86 | 0.001 |
|  | Sep/Div | 0.54 | 0.40-0.72 | <0.001 | 0.68 | 0.50-0.93 | 0.014 |
|  | Widowed | 0.88 | 0.68-1.15 | 0.342 | 0.70 | 0.52-0.93 | 0.014 |
| Education | Primary | 1.00 | Ref |  | 1.00 | Ref |  |
|  | Secondary | 1.13 | 0.96-1.33 | 0.141 | 1.30 | 1.08-1.56 | 0.005 |
|  | Third Level | 1.57 | 1.31-1.89 | <0.001 | 1.49 | 1.21-1.83 | <0.001 |
| Employment | Employed | 1.00 | Ref |  | 1.00 | Ref |  |
|  | Retired | 1.47 | 1.26-1.72 | <0.001 | 1.23 | 0.99-1.53 | 0.056 |
|  | Other | 0.53 | 0.42-0.64 | <0.001 | 0.67 | 0.53-0.86 | 0.002 |
| Smoking Status | Never | 1.00 | Ref |  | 1.00 | Ref |  |
|  | Past | 1.06 | 0.90-1.24 | 0.501 | 0.97 | 0.82-1.15 | 0.746 |
|  | Current | 0.45 | 0.37-0.55 | <0.001 | 0.56 | 0.46-0.69 | <0.001 |
| Number of GP visits | Continuous | 1.03 | 1.02-1.05 | <0.001 | 1.03 | 1.01-1.05 | 0.001 |
| Influenza Vaccine | Ever | 1.72 | 1.48-1.97 | <0.001 | 1.35 | 1.14-1.60 | 0.001 |
| Chronic illnesses | Continuous | 1.22 | 1.16-1.30 | <0.001 | 1.11 | 1.04-1.18 | 0.001 |
| Prior Cancer diagnosis |  | 1.78 | 1.13-2.82 | 0.013 | 1.48 | 0.91-2.40 | 0.112 |
| Treated BPH |  | 3.67 | 2.31-5.82 | <0.001 | 2.64 | 1.64-4.25 | <0.001 |
| GMS Scheme Eligible |  | 0.84 | 0.73-0.96 | 0.014 | 0.64 | 0.52-0.78 | <0.001 |
| No of medicines |  | 1.11 | 1.08-1.14 | <0.001 |  |  |  |
| Private Health Insurance |  | 2.34 | 2.03-2.71 | <0.001 |  |  |  |
| Cholesterol test |  | 16.33 | 12.4-21.6 | <0.001 |  |  |  |

Multivariate OR is adjusted for age (continuous), marital status (married/ single/separated or divorced/ widowed), education level attained (primary/ secondary/ third level), employment status (employed/retired/other), smoking status (never/past/ current), number of GP visits in the past year (continuous), receipt of influenza vaccine (ever/never), number of chronic illness reported (continuous), prior cancer diagnosis (excluding other than prostate cancer), GMS eligibility (yes/no) and reported receipt of medicines for BPH.
